# Evaluating the skill of AI and physics-based models for February 2020 North Atlantic storm hazards

**DOI:** 10.1038/s44304-026-00223-6

**Published:** 2026-05-27

**Authors:** Hilla Afargan-Gerstman, Rachel W.-Y. Wu, Alice Ferrini, Daniela I. V. Domeisen

**Affiliations:** 1 Oeschger Centre for Climate Change Research, Institute of Geography, University of Bern, Bern, Switzerland; 2 Institute for Atmospheric and Climate Science, ETH Zurich, Zurich, Switzerland; 3 Department of Applied Physics and Applied Mathematics, Columbia University, New York, NY USA; 4 Faculty of Geosciences and Environment, University of Lausanne, Lausanne, Switzerland

**Keywords:** Climate sciences, Natural hazards

## Abstract

The emergence of data-driven weather forecast models provides great promise for producing faster, computationally cheaper weather forecasts, compared to physics-based numerical models. However, while the performance of artificial intelligence (AI) models has been evaluated primarily for average conditions and single extreme weather events, less is known about their ability to capture sequences of extreme events – periods that are usually accompanied by multiple natural hazards. The February 2020 storm series provides a prime example to evaluate the performance of AI models for predicting multiple storm hazards. This event was associated with severe surface impacts including intense surface wind speeds and heavy precipitation, amplified regionally due to the close succession of three extratropical storms. In this study, we compare the performance of data-driven models to physics-based models in forecasting the February 2020 storm series over the United Kingdom. Our results show that, for these case studies, AI models tend to outperform the numerical model in predicting mean sea level pressure (MSLP) on weekly timescales, and, to a lesser extent, surface winds. Nevertheless, certain ensemble members within the physics-based forecast system can perform as well as, or occasionally outperform, the AI models. Moreover, weaker error correlations between atmospheric variables suggest that AI models may overlook physical constraints. This analysis helps to identify gaps and limitations in the ability of data-driven models to be used for multi-hazard warnings, and emphasizes the need to integrate such models with physics-based approaches for reliable impact forecasting.

## Introduction

The recent emergence of artificial intelligence (AI) provides new pathways for producing weather forecasts in less time and at lower computational cost^1,2^ However, there is a need to evaluate the capability of these models to reproduce extreme events and their associated surface impacts at the regional scale, especially in the context of weather extremes that occur in close succession, for instance recurrence of extratropical storms, which often leads to compounding effects of damaging winds and flooding.

Data-driven forecasts have been compared to physics-based forecasts for single storm events^3,4^, and in systematic assessments of their performance^3,5^ analyzed this aspect by comparing forecasts based on data-driven versus physics-based Numerical weather prediction (NWP) models for the Storm Ciarán, which hit Europe in November 2023. They found that data-driven forecasts, despite accurately reproducing the synoptic-scale structure of the storm, failed to accurately estimate the wind speed. For the 2021 North American winter storm^4^, found that the data-driven models performed comparably or better than the physics-based forecast, particularly in forecasting compound winter storm conditions (including wind speed, temperature and wind chill). More recently^5^ have used feature-based tracking to evaluate the performance of NWP models and data-driven weather prediction models in forecasting midlatitude cyclones at 10-day forecast lead times, showing that data-driven models systematically underestimate cyclone minimum sea level pressure (MSLP), compared to physics-based forecasts, with weaker maximum 10-m winds than in the observations.

Other studies have evaluated the performance of data-driven weather prediction models in forecasting weather extremes 10 days in advance^4,6^. According to these studies, data-driven models were found to perform as good as the deterministic forecast of the physics-based model for near-surface temperature and wind extremes, however their performance varies by region and forecast lead time^6^. Furthermore, while data-driven models can reach similar accuracy to ECMWF’s NWP model at the local scale, their performance is lower when variables are aggregated over space and time^4^.

However, despite recent progress in forecasting of extreme weather events, a critical gap remains in understanding how data-driven models perform during a sequence of weather systems, for instance, when multiple storms occur in the same region within a short time span. Storm clustering poses an increased risk to physical infrastructure and human life, as the same regions are repeatedly hit by strong winds and large amounts of precipitation^7–9^. Consequently, the ability of models to capture the evolution of such a sequence of events, rather than focusing on a single event, is essential for accurately assessing damage potential and improving early warning systems.

Such a close succession of storms occurred in February 2020, when the three cyclones Ciara, Dennis, and Jorge hit the United Kingdom within a short time period. Such conditions tend to result in a shorter recovery time between the events, often leading to serious socioeconomic consequences such as flooding of rivers, disruption of transportation, and damage to infrastructure.

On February 8, Storm Ciara (also known as Sabine, Elsa) hit the United Kingdom (UK), bringing windy weather with persistent heavy rain, especially over northwestern England. A week later, on February 15, Storm Dennis (also known as Victoria), one of the deepest Atlantic depressions on record^10^, impacted the British Isles and north-western Europe and brought even wetter conditions, prompting the UK Met Office to issue red weather warnings for part of South Wales. While wind speed and tidal surges during storms Ciara and Dennis were substantially higher than the February mean, precipitation exhibited the largest anomaly, leading to extensive impacts over Western Britain^11^. Finally, on February 28, Storm Jorge hit the UK, which, although the least intense of the three, Storm Jorge added to the extremely prolonged period of rainfall ^12,13^ and contributed to worsening the overall damage already caused by Ciara and Dennis.

Overall, the meteorological conditions in February 2020 led to exceptionally heavy rainfall across the United Kingdom, making it the wettest February on record for many regions^10,12,13^. River flows responded rapidly to this rainfall, as soils were already near saturation from preceding precipitation events, resulting in record river discharges and extended flooding. The subsequent river floods caused severe damage across Wales, northern England, and the Midlands. The total insured losses from the February 2020 UK floods were evaluated at approximately GBP 368 million^14^. Specifically, the total industry loss for storms Ciara and Dennis (including damage over the British Isles and Continental Europe) was estimated at EUR 1571 million and EUR 350 million, respectively^15,16^.

Data-driven forecasts have demonstrated improved performance on short-term predictions ^17,18^ as well as medium range forecasts (up to 2 weeks in advance) for various atmospheric variables (including temperature and wind) and their extremes^1,4,6,19–22^. However, forecasting a series of storms, rather than a single event, on weekly timescales can be a challenging task^7^. On these subseasonal timescales, extratropical cyclone clustering is modulated by both local and remote drivers; Clustering may depend on the properties of the primary cyclone and the local conditions in which it develops, while remote drivers, such as sea surface temperature anomalies and stratospheric variability, can influence the subsequent storm development and their downstream propagation. Specifically, unusually strong stratospheric polar vortex conditions may increase the likelihood of intense extratropical cyclones impacting the UK^23^, which was also shown for the storm clustering over the UK in February 2022^24^. Assessing model performance for a sequence of natural hazards such as storm clustering or storm series events, is an essential step towards increased reliability of impact predictions by data-driven models as storms may lead to increased damage when temporally and spatially aggregated. Therefore, model verification for these events can help to advance their potential use for socioeconomic preparedness and early warnings.

This study investigates the performance of data-driven weather prediction models in capturing the wind hazards associated with extratropical storms over the North Atlantic and Europe through three representative case studies occurring in close succession. Specifically, we compare two distinct modeling approaches: AI-based architectures trained on ERA5 data without explicit physics, and traditional physical models that rely solely on dynamical equations without prior training, in their ability to represent the storm clustering event, and evaluate forecast error correlations between physically-linked variables.

## Methods

### Weather forecast models

In this study, we evaluate the performance of medium-range forecasts by a physics-based model and two data-driven model against reanalysis data. All datasets are obtained from WeatherBench 2, an open source evaluation framework for medium-range global weather forecasting that provides datasets on Google Cloud Storage with a time step of 6 hours at a resolution of 0.25° or higher^1^.

We focus on three initialization dates: February 1, 8, and 21 (referred to as the ”forecast initialization date”). For each initialization date, the forecast is validated for a window up to a lead time of 10 days. Lead time is defined as the time interval between the initialization day and the day for which the forecast is validated (”valid time”). The initialization dates are selected such that the peak of storm intensity of the storms of interest (Ciara, Dennis, and Jorge) occurs on the 8th lead day of each forecast.

#### Numerical weather prediction model

Numerical weather prediction (NWP) models solve sets of mathematical equations for the atmosphere and oceans to create a prediction of the weather based on current weather conditions. Here, we use 50-member operational ensemble forecasts of the European Centre for Medium-Range Weather Forecasts (ECMWF) Integrated Forecasting System (IFS). These ensemble forecasts were retrieved from the THORPEX Interactive Grand Global Ensemble (TIGGE) archive^25^ through WeatherBench 2. IFS ensemble mean (IFS ENS mean) is computed by taking an average over the 50 members and is used as a baseline in ref. ^26^ as it performs well on deterministic error metrics. The model version used here belongs to the IFS cycle 46r1.

To compare to the data-driven models, which are deterministic, a best-performing member is chosen from each forecast initialization by selecting the member with the smallest Mean Absolute Error (MAE) (see the Methods Section for details on the MAE computation).

#### Data-driven models

With the recent advancements in artificial intelligence, it has been possible to expand the number of forecast datasets by including data-driven weather forecasting models. Here we evaluate two data-driven models: Pangu-Weather^27^, developed by Huawei and based on a three-dimensional Earth-specific transformer and hierarchical temporal aggregation; and GraphCast^19^, developed by Google DeepMind and based on graph neural networks.

Data-driven models typically follow a three-phase development process: training, validation, and prediction (testing). Specifically, Pangu-Weather is trained in a first phase on past data, in this case, the European Centre for Medium-Range Weather Forecasts (ECMWF) ERA5 reanalysis data^28^ from January 1979 to December 2017. A similar process is used for GraphCast, which involves, however, training on ERA5 data from January 1979 to December 2019. Both Pangu-Weather and Graphcast used in this study are deterministic. Probabilistic weather forecasting models, such as AIFS^29^ and GenCast^20^ provide machine-learned ensemble forecasts that are competitive with physics-based NWP models for a range of across probabilistic forecast scores; however, available AIFS forecasts start later than February 2020, and therefore for this study only deterministic ("single") forecasts were used.

### Forecast verification

The evaluation of the physics-based and AI models is performed on the three cyclones that have occurred in close succession in the North Atlantic sector in February 2020: Ciara, Dennis and Jorge. We evaluate the forecasts for two main variables that are directly relevant to storm impacts, namely mean sea level pressure (MSLP) and 10-m wind speed, which correspond to storm intensity and the associated surface impact. All model output is validated against ERA5 reanalysis^28^ at a 1.5° horizontal resolution as obtained from WeatherBench 2^1^. While the AI weather models are trained on the ERA5 reanalysis dataset, the events and time periods we evaluated in this study were outside the training phases of the models to ensure robust assessments of the forecasts^1^.

Here, we use 6-hourly data at 1.5° spatial resolution, averaged to daily means for specific analyses. We focus the analysis on the forecasts initialized at 00 UTC. Anomalies of these variables are computed as deviations from their daily climatological mean from 1990 to 2019, obtained from ERA5 reanalysis.

**Fig. 1:**
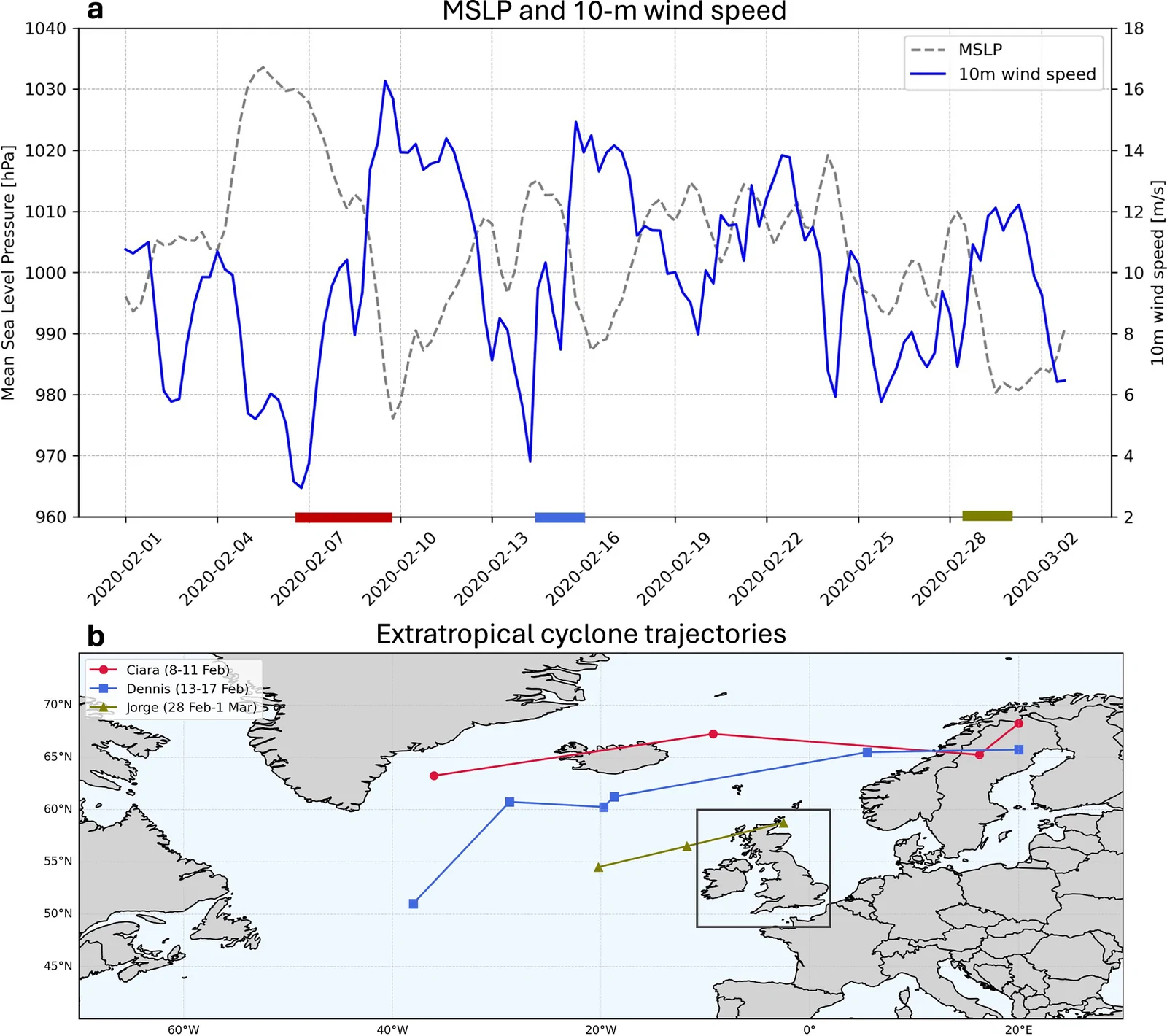
Time series of MSLP and 10-m wind speed during the February 2020 storm series. **a** Time series of MSLP (dashed grey line) and 10-m wind speed (solid blue line) over the UK (12°W-5°E, 48°N-60°N; black box in (**b**)) during the storm series in February 2022. (b) Trajectories of the three storms (Ciara, Dennis and Jorge) over the North Atlantic and Western Europe, based on daily minimum MSLP anomalies computed relative to the daily 30-year climatology (see the Methods section for details).

In order to assess the reliability of the forecasts for storm hazards, we choose the UK as our study region (48–60°N, 12°W–5°E; Fig. 1), a region that was heavily impacted by the successive passage of the storms in February 2020. All variables evaluated in the study are averaged over the above study region.

To quantify the error in forecasting cyclone intensity in terms of MSLP and wind speed, the Mean Absolute Error (MAE) is calculated as:

1 $$\,\mathrm{MAE}\,=\frac{1}{N}\mathop{\sum }\limits_{i=1}^{N}| {F}_{i}-{O}_{i}|$$

where *F*_*i*_ denotes the forecasted value and *O*_*i*_ the observed value, both at time i. The variable N represents the number of time steps. MAE is useful for comparing the average magnitude of the forecast errors, regardless of their sign (i.e. overestimation or underestimation). A perfect forecast would result in an MAE of 0.

### Storm severity index

To quantify the potential destructive power of an extratropical cyclone, the Storm Severity Index (SSI) is used^30^. The SSI metric identifies high-impact episodes relative to the local climatology. The index is defined as follows:

2 $$SSI={\left(\frac{V}{{V}_{ref}}-1\right)}^{3}$$

where *V* is the local 10 m wind speed and *V*_*ref*_ is a constant threshold set at 20 m/s. This value corresponds to the onset of the Beaufort 9 threshold (Strong Gale), which is generally associated with structural damage and uprooted trees in mid-latitude environments. The use of a cubic power reflects the empirical relationship between wind speed and damage^30–33^.

## Results

### The storm series in February 2020

In this section, we present a comparison between the physics-based weather prediction model and the data-driven models, with aim of quantifying the models’ ability to reproduce the characteristics of the observed storm clustering event, as well as their ability to maintain the correlations between physically-linked variables. We focus on the forecast verification of a series of extratropical storms over the UK in February 2020.

We analyze storms Ciara, Dennis, and Jorge, which hit the UK in rapid succession in February 2020. Although this event is not defined as a storm clustering event in the literature^10^, it nevertheless featured a close temporal succession of the three storms over the northern part of the North Atlantic and the UK, with intensity peaks on 8-9 February for Storm Ciara, 15-16 February for Storm Dennis, and 28–29 February for Storm Jorge. Similar close succussions of cyclones over Western Europe were recorded in several past winters, including 1990, 1993, 1999, 2007, and 2014, e.g., refs. ^7,30,34^.

As a first qualitative assessment, we analyze the timeseries of MSLP (dashed) and 10m wind speed (in blue) observed over the UK region during February 2020 (Fig. 1a), and their corresponding cyclone trajectories in the North Atlantic basin: Ciara (in red), Dennis (in blue), and Jorge (in green) (Fig. 1b). Storm Ciara developed rapidly on February 7 (but was particularly noteworthy already on February 4) reaching its maximum intensity one day later between Iceland and Greenland, with a strongly negative MSLP anomaly of -60 hPa with respect to climatology over the North Atlantic, and wind speed anomalies reaching 15 m/s (Fig. 1a). The cyclonic conditions brought intense northwesterly winds towards the UK, recording one of the strongest wind anomalies of this month in the region (10 m/s), accompanied by a drop in MSLP (-45 hPa). The cyclone then gradually weakened over the following days as it moved towards Scandinavia. Storm Ciara was characterized by an exceptionally wide area that was affected by damaging wind gusts across the British Isles and Continental Europe^15^.

A second cyclone, Storm Dennis, formed on 15 February in the middle North Atlantic and rapidly intensified as it moved northeastward. It reached its peak intensity on February 16, recording the most intense pressure drop of the month, with an MSLP anomaly of -65 hPa and a maximum wind speed anomaly of 18 m/s (Fig. 1a). The winds associated with Dennis extended across a large area of the North Atlantic and Western Europe, significantly affecting the UK, where maximum wind speed anomalies (11 m/s) and the strongest negative minimum MSLP anomaly (-55 hPa) for February in this region have been recorded. In the following days, Dennis gradually lost intensity as it approaches Norway, dissipating completely by February 20. Storm Jorge developed on 28 February to the west of the UK (Fig. 1a) and within a few hours reaches a MSLP anomaly of -50 hPa and a wind speed anomaly of 13 m/s northwest of Ireland. Despite being the least intense of the three cyclones over the North Atlantic, Storm Jorge had comparably strong UK wind anomalies. Finally, the storm weakened and dissipateed in the first days of March.

### Forecast verification of storm intensity and surface winds

**Fig. 2:**
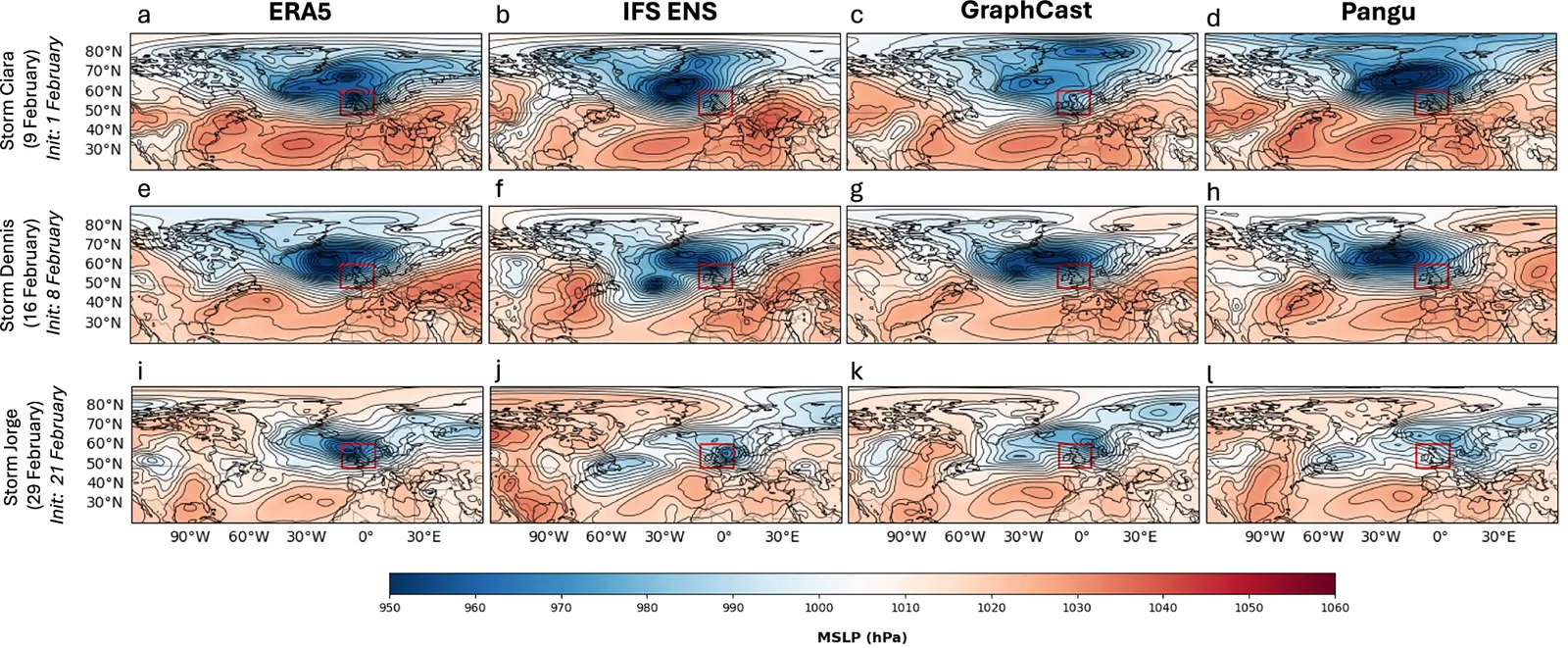
Evaluating MSLP of storms Ciara, Dennis, and Jorge over the North Atlantic and Western Europe in physics-based and AI models. MSLP (shading) over the North Atlantic and Western Europe (20–80°N, 60°W–20°E) for the days of peak storm intensity: **a**–**d** Storm Ciara on 9 February (initialization on 1 February), **e**–**h** Storm Dennis on 16 February (initialization on 8 February) and **i-l** Storm Jorge on 29 February (initialization on 21 February). Data are derived from (from left to right): ERA5 reanalysis, IFS ENS mean, GraphCast, and Pangu-Weather. The study region over the UK is indicated by the red box.

**Fig. 3:**
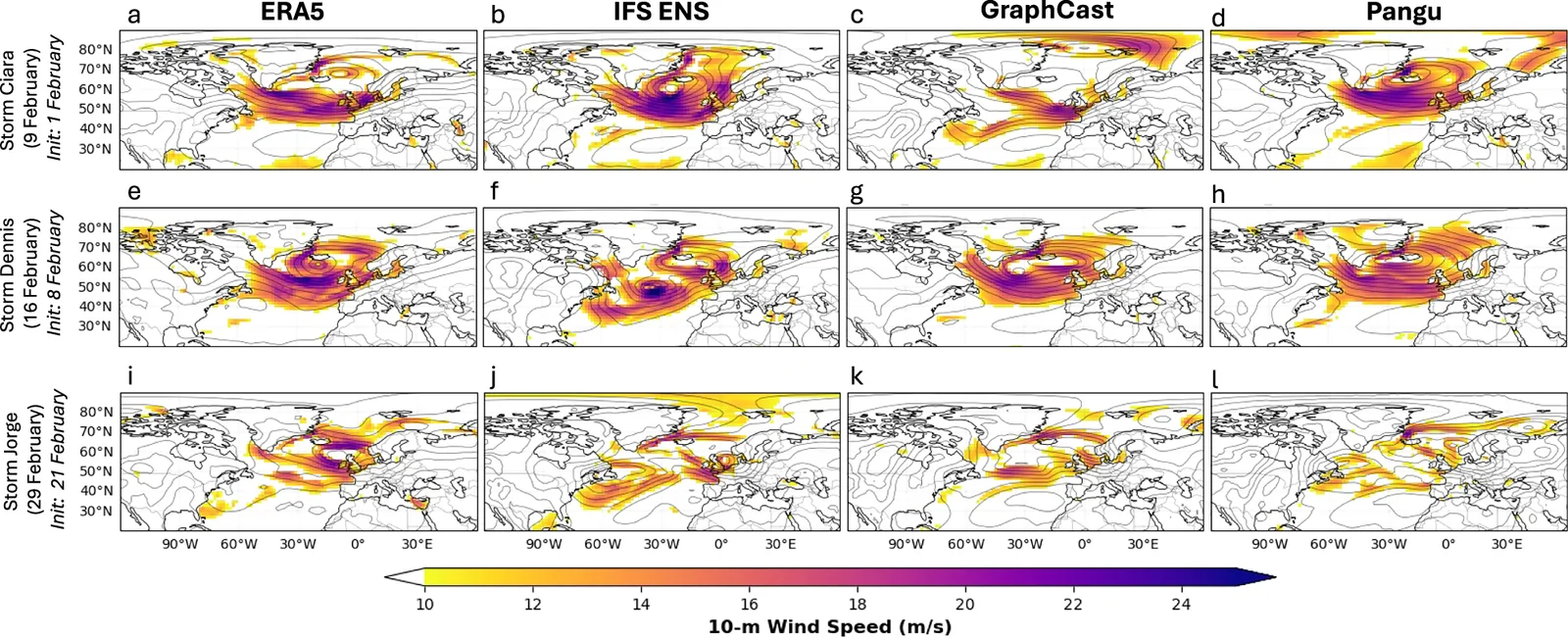
As Fig. 2 but for surface wind speed of storms Ciara, Dennis, and Jorge over the North Atlantic and Western Europe in physics-based and AI models. 10 m wind speed (shading) over the North Atlantic and Western Europe (20–80°N, 60°W–20°E) for the days of peak storm intensity: **a**–**d** Storm Ciara on 9 February (initialization on 1 February), **e**–**h** Storm Dennis on 16 February (initialization on 8 February) and **i**–**l** Storm Jorge on 29 February (initialization on 21 February). Data are derived from (from left to right): ERA5 reanalysis, IFS ENS mean, GraphCast, and Pangu-Weather.

Figures 2 and 3 show the MSLP (shading) and 10-m wind (shading), respectively, for the three storms (Ciara, Dennis, Jorge) on their day of maximum development, comparing reanalysis (ERA5) with NWP model forecasts (IFS ENS mean) and the two data-driven models (GraphCast and Pangu-Weather), at lead times of 8 days with respect to the forecast initialization date (see Figure labels for the dates).

Overall, all models are able to capture realistic storm structures at their peak magnitudes (Figs. 2 and 3), although the accuracy in capturing both spatial structure and magnitude varies between specific storm events, even within the same storm series. For storms Ciara and Dennis (panels a-d and e-h in Figs. 2 and 3), both physics-based and data-driven models show agreement in representing the cyclone intensity and structure. However, for Storm Jorge, data-driven models tend to underestimate the peak magnitudes of both MSLP and surface wind speeds.

Generally, GraphCast and Pangu-Weather predict MSLP and wind speed magnitudes that are more comparable to ERA5 than the IFS ENS mean, which tends to underestimate both variables across the series. However, capturing storm intensity in terms of MSLP does not necessarily translate into an equally accurate forecast of the surface wind speed, particularly during the peak of Storm Jorge (see also Table 1).

**Table 1:** Mean absolute error (MAE) for MSLP and 10-m surface wind, averaged over the UK, for all storm events and all models

Storm	IFS EM	IFS best member	GraphCast	Pangu-Weather	Mean
**MSLP MAE [hPa]**					
Storm Ciara Storm Dennis Storm Jorge **Mean (all storms)**	5.46 5.52 6.83 5.94	4.24 3.59 5.55 4.46	2.07 4.15 3.45 3.23	4.40 4.40 5.20 4.66	3.98 4.69 5.16 4.61
**Wind MAE [m/s]**					
Storm Ciara Storm Dennis Storm Jorge **Mean (all storms)**	0.99 1.09 1.61 1.23	0.77 0.64 1.02 0.81	1.57 0.87 1.26 1.24	1.00 1.01 1.36 1.12	1.19 0.99 1.41 1.20

MAE is computed using 6-hourly forecast data initialized at 00:00 UTC. ’IFS EM’ denotes the ensemble mean, and ’IFS best member’ refers to the best-performing ensemble member for a 10-day forecast. The rightmost column represents the average of IFS EM, GraphCast, and Pangu-Weather.

**Fig. 4:**
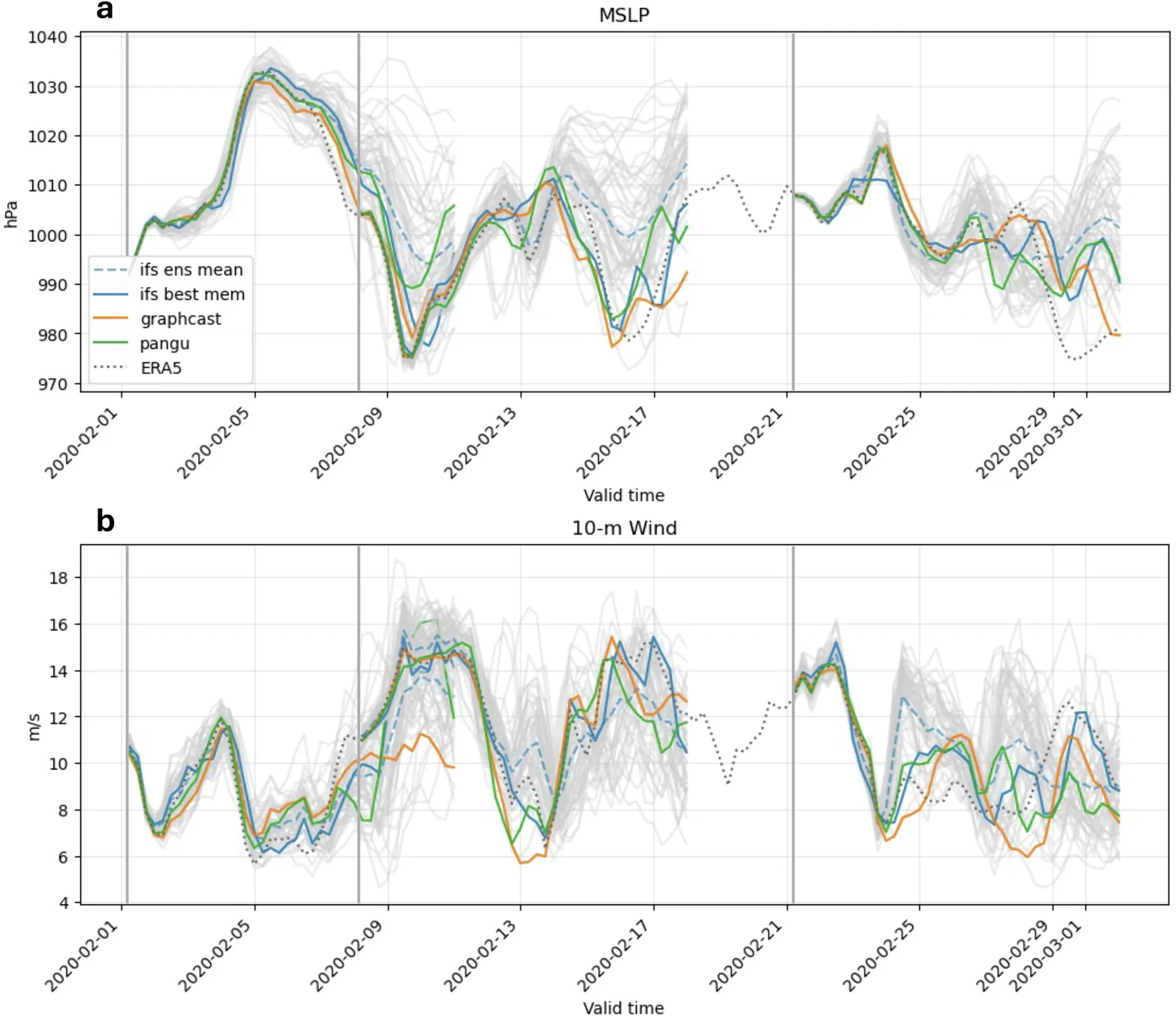
Physics-based vs. AI models: predicted timeseries over the UK. Predicted timeseries of (**a**) MSLP and (**b**) 10 m wind averaged over the UK for the forecast models for the three initialization dates: 1 February (Storm Ciara), 8 February (Storm Dennis), and 21 February (Storm Jorge). Forecasts are plotted for three models: the physics-based model IFS (blue solid line) and two AI models: Graphcast (orange) and Pangu (green). IFS ensemble members are plotted in solid grey lines, and the ensemble mean is plotted in dashed blue. The best member of IFS in each initialization is highlighted in solid blue line. The ERA5 is plotted in dotted grey line. Vertical grey solid lines indicate dates of forecast initialization.

Figure 4 further compares the evolution of MSLP (in hPa) and surface wind speed (in ms^−1^) between the forecasts (colored lines) and against ERA5 (dotted grey line) up to 10 days after initialization. Each graph displays the three initialization times, matching each of the storms: Ciara (February 1), Dennis (February 8), and Jorge (February 21).

At short lead times, all models produce MSLP and winds that closely match the observed values. However, as lead time increases, distinct differences emerge between the data-driven forecasts and the deterministic baseline of physics-based model. The IFS ENS mean (dashed blue line) captures the overall timing of each cyclone’s deepening over the UK but simulate weaker cyclones, underestimating the MSLP minima of Ciara and Dennis by approximately 20 hPa and Jorge by about 25 hPa. As the cyclones decay, the MSLP in IFS ENS mean forecasts gradually weakens, following the observed dissipation rate. In contrast, the data-driven models (orange and green lines for GraphCast and Pangu-Weather, respectively) predict magnitudes of MSLP and wind speeds closer to ERA5 (Fig. 4).

Yet, it is important to notice that while IFS ENS mean may underestimate the intensities of the cyclones and the associated surface wind, we can find IFS ENS ensemble members that accurately predict the observed deepening and decay rates of the cyclone series (solid grey line). The best-performing member (solid blue line) is indicated in Fig. 4 for each of the cyclone events. The best-performing member here is selected as the one with the smallest MAE in 10-m wind speed, averaged over all lead times. The evolution of the best-performing member is found to be similar to that of the AI models.

**Fig. 5:**
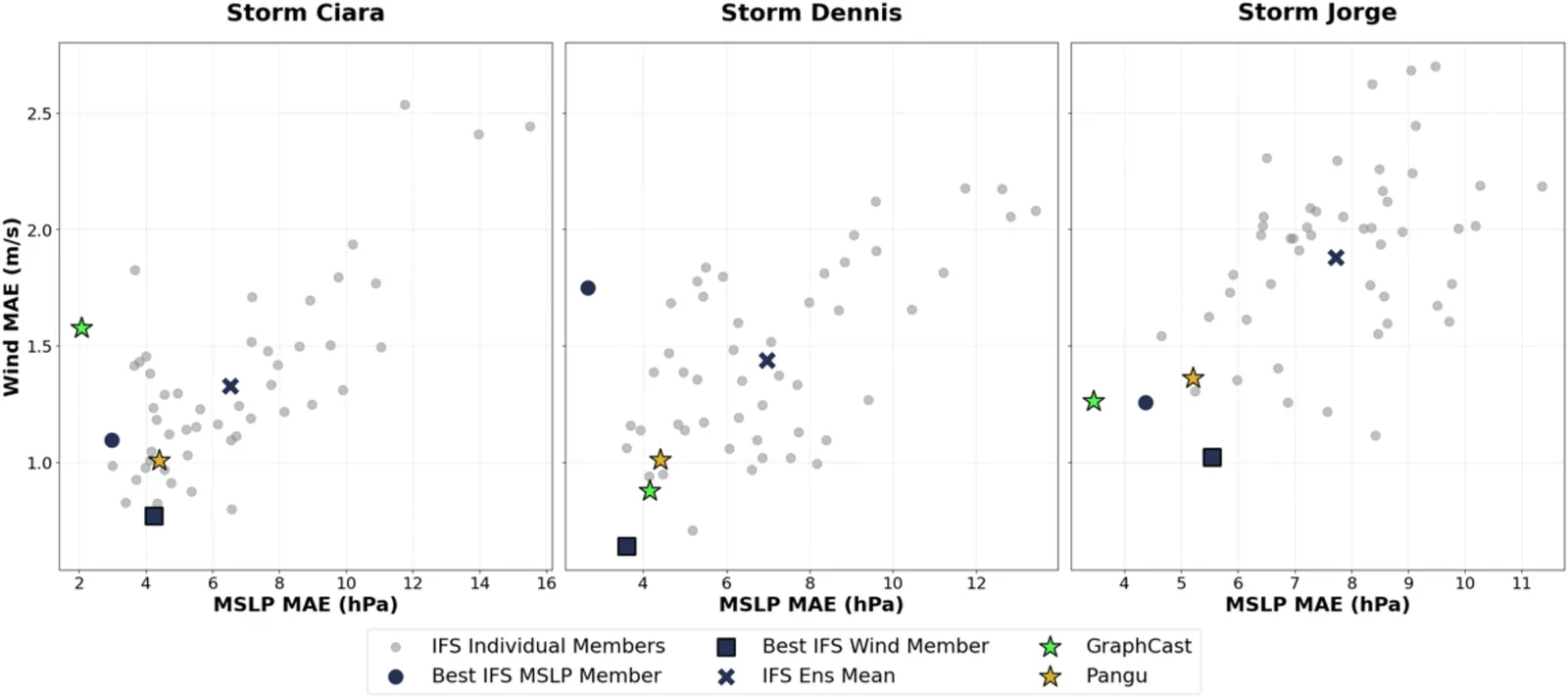
Surface wind and MSLP mean absolute errors at the time of peak storm intensity of storms Ciara, Dennis and Jorge. Relationship between MAE for 10m wind speed and MSLP computed using 6-hourly data (UK average; see Fig. 1). Data spans three model initializations for Storms Ciara, Dennis, and Jorge. Individual IFS ensemble members are represented by grey circles, with the ensemble mean denoted by 'x'. AI model performance is marked by stars (orange: GraphCast; green: Pangu-Weather). The best-performing IFS members for both wind and MSLP are indicated in black (square: wind; circle: MSLP).

Figure 5 demonstrates the forecast verification for MSLP and 10 m wind using MAE values for each storm event separately. In predicting MSLP, GraphCast is the most skillful model with averaged MAE of 3.23 hPa, followed by IFS best member, Pangu-Weather, and IFS ENS mean. The best member of IFS is most skillful in predicting the 10 m wind among all models, with the lowest average MAE of 0.81 ms^−1^, followed by Pangu-Weather, GraphCast, and IFS ENS mean. Fig. 5 highlights that the best member of IFS is not the same for both storm variables (Fig. 5). Table 1 summarizes MAE values for each storm event, and on average for all storms.

Overall, out of the three storms in the February 2020 series, Storm Ciara is the best predicted storm by the models in MSLP with averaged MAE of 3.98 hPa. In terms of surface wind speed, Storm Dennis is the best predicted, with averaged MAE of 0.99 ms^−1^ over all models.

### Sources of forecast bias

**Fig. 6:**
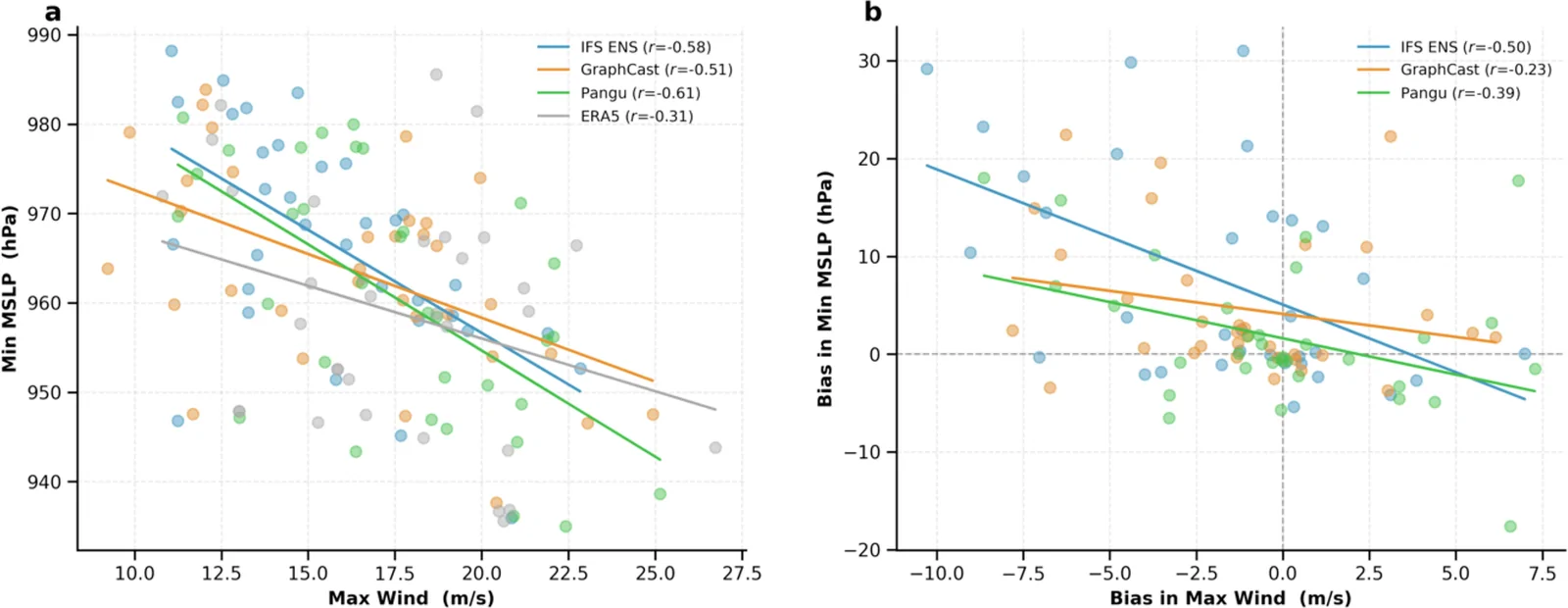
Evaluating the correlation between MSLP and wind values and errors across models and lead times. **a** Scatter plot of daily values for the minimum MSLP and the maximum 10m wind speed within a 6° × 6° box centered at the cyclone MSLP minimum, averaged across all initializations. Each dataset is represented by a different color: grey for ERA5 observations, blue for IFS ENS mean, orange for GraphCast, and green for Pangu-Weather. A linear regression line is fitted to each model’s dataset, with the corresponding correlation coefficient (*r*) indicated in the legend. **b** The relationship between maximum 10m wind speed bias and the minimum MSLP bias, within a 6° × 6° box centered at the cyclone MSLP minimum.

Understanding the causes and sources of forecast bias in data-driven and dynamical models requires assessing the way these models represent physical constraints. For this purpose, we visualize the relationship between the minimum MSLP of each storm at each lead time of the forecast, and the associated maximum surface wind speed (Fig. 6a), as well as their respective errors (Fig. 6b). Figure 6a shows the relationship between MSLP and the surface wind within a 6° × 6° box centered at each cyclone MSLP minimum.

Each point in Fig. 6a indicates the individual daily values averaged across all three initializations, and the colors are used to distinguish the different forecast models and observations. For each dataset, a regression line is then fitted to quantify the strength and sign (positive or negative) of the relationship between these two variables, and the corresponding correlation coefficient (r) is calculated. Overall, a negative correlation is observed, indicating that low MSLP values are associated with high wind speed values. IFS ENS mean exhibits a strong correlation coefficient (*r* = −0.58) between minimum MSLP and maximum wind speed values, while the data-driven models show a weaker relationship, especially for GraphCast (r=-0.51) (Fig. 6a). Compared to the observations, which exhibit a correlation coefficient of *r* = −0.31, IFS shows a stronger inverse relationship between MSLP and wind speed, potentially overestimating this correlation.

Next, we examine the error correlation for minimum MSLP and maximum surface wind for all three initializations (Fig. 6b). Unlike physics-based models, AI models show weaker error correlations between physically linked variables, such as storm intensity and surface wind. Both GraphCast and Pangu-Weather show a weaker correlation compared to IFS, with *r* = -0.23 and *r* = -0.39, respectively. In comparison, IFS shows a relatively strong negative correlation, with *r* = -0.50. These results indicate that for the AI models, storm intensity forecasting errors (as measured e.g., within a box centered at the cyclone MSLP minimum) do not necessarily translate into errors in surface wind prediction.

### Forecasting storm severity in physics-based and AI models

**Fig. 7:**
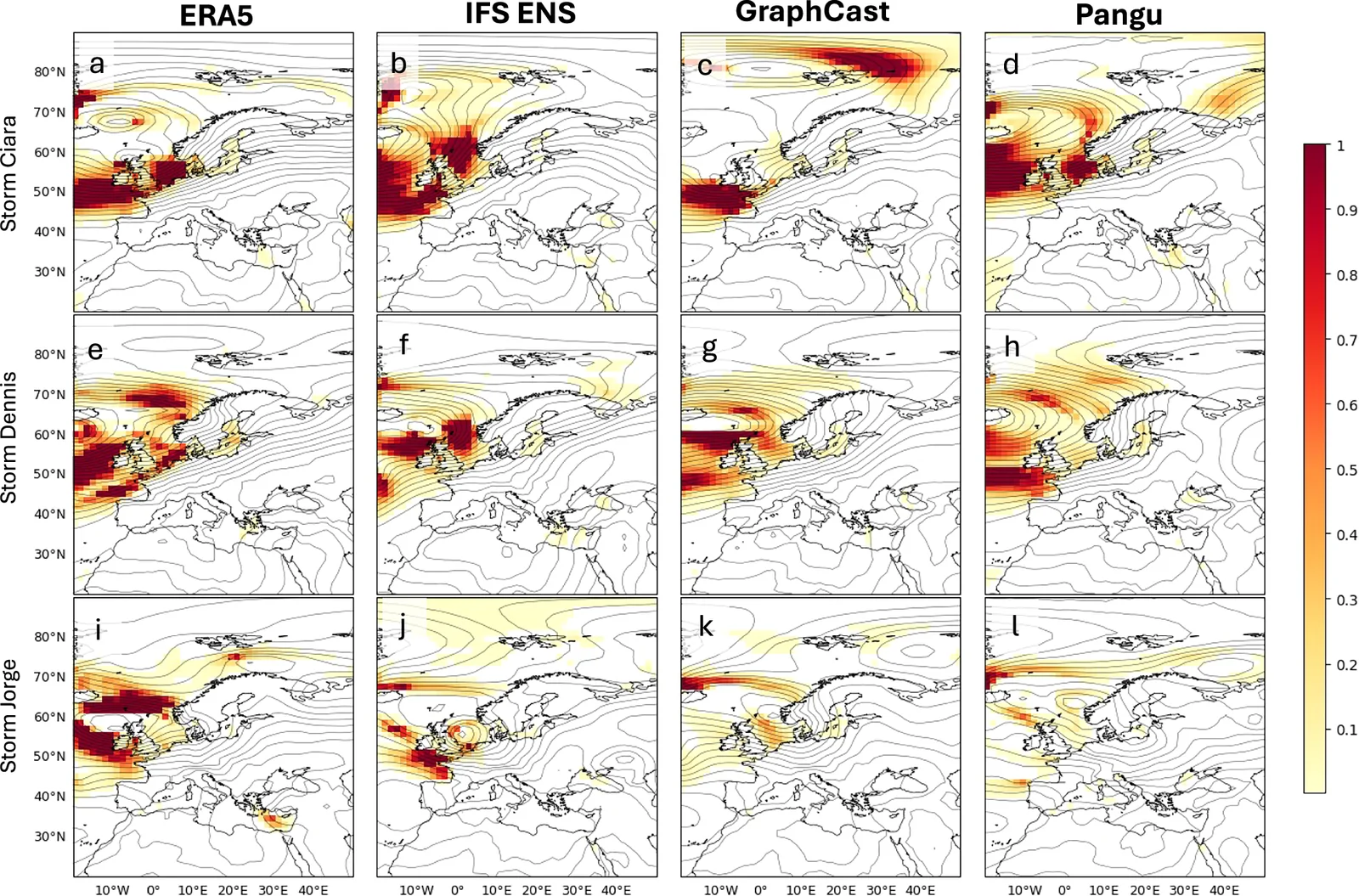
Storm severity index in physics-based and AI models for the days of peak storm intensity. Storm severity index (SSI; shading) for the days of peak intensity for Storm Ciara on 9 February (**a**–**d**; initialization on 1 February), Storm Dennis on 16 February (**e**–**h**; initialization on 8 February), and Storm Jorge on 29 February (**i**–**l**; initialization on 21 February). Data is derived from (from left to right): ERA5 reanalysis, IFS ENS mean, GraphCast, and Pangu-Weather. SSI values are non-dimensional and scaled by 10^−2^.

Here, we investigate how well physics-based and AI models represent storm severity in forecasts of the February 2020 storms. We use the SSI metric as a measure of the severity of the storms from a meteorological perspective. Figure 7 shows the SSI over the Euro-Atlantic region at the time of peak storm intensity for Storms Ciara, Dennis, and Jorge. The magnitude of the SSI in these case studies is found to be underestimated in both physics-based and AI models, consistent with the 10-m wind field.

## Discussion

This study performs a comparison between physics-based and data-driven weather prediction models: ECMWF’s IFS ENS mean, GraphCast, and Pangu-Weather, in forecasting a series of extratropical cyclones that hit the UK in February 2020. The ability of a weather model to predict the intensity of each storm in the storm series in terms of MSLP, wind speed, and their location with a high degree of accuracy is critical to support effective disaster preparedness, addressing an increasingly complex challenge due to their aggregated impacts^23,24^. In this context, the rapid evolution of the forecasting capabilities of AI models represents, on the one hand, a great opportunity for atmospheric sciences and, on the other, a significant challenge, as it is still unclear how well these models can reproduce extreme storm hazards and predict their impacts at a regional scale.

Our results show that both data-driven and physics-based models tend to underestimate storm intensity (MSLP) and surface wind intensity, particularly at lead times beyond 5–7 days. The data-driven models (in this study, GraphCast and Pangu-Weather) show comparable or better skill as compared to the ensemble mean of the physics-based model in reproducing surface winds associated with extratropical cyclones (Fig. 5; Table 1).

In addition, unlike the physics-based model, the data-driven models show weaker error correlations between physically linked variables, such as storm intensity (measured by the minimum MSLP of the storm) and surface wind, indicating an improved ability of data-driven models in predicting the surface wind field (and consequently, windstorm-related impacts), yet while potentially misrepresenting physical constraints. Hence, at current, predicting impacts from data-driven models remains challenging given the limited explainability of AI model biases. Specifically, the question arises if an AI-based forecast of high surface wind speeds can be trusted for an impact-based warning if the associated storm is poorly resolved, misplaced, or incorrectly predicted in the data-driven model. As data-driven models are optimized to minimize error metrics, this can explain their smoother storm structure and reduced variability.

Additionally, while ERA5 provides globally consistent wind fields, it exhibits limitations in capturing near-surface wind extremes due to the representation of sub-grid scale processes at a 0.25° horizontal resolution. Therefore, wind speeds likely represent the lower bound of peak intensities. These limitations suggest that the training data used by the data-driven models may be inherently biased toward moderate values. Furthermore, analyzing daily means may smooth localized extremes, yet it is suitable when considering medium-range or sub-seasonal to seasonal lead times.

At the same time, our study demonstrates that while the ensemble mean of IFS overall has weaker prediction skill as compared to the data-driven models, there are single ensemble members within IFS that outperform the data-driven models. Therefore, given the deficient physical consistency found for the February 2020 storm series, AI models (in their current state) can be recommended for use for impact warnings only with access to a prediction from a physical model at the same time. In fact, given the high skill of single ensemble members, it might at current still be more advisable and lead to more trustworthiness to use data-driven methods to help select the best performing ensemble member from the physical model. Ensemble sub-selection methods, such as those outlined in ref. ^35^ and applied in ref. ^36^ for seasonal prediction based on simple statistical methods, may potentially be applied to short- and extended-range forecasts using data-driven approaches for performing the sub-selection. Similar methods of combining physical and data-driven models exist within so-called hybrid forecasting methods for predicting impacts e.g., refs. ^37,38^.

A promising direction for improving storm prediction would be to extend the analysis to additional storm clustering episodes, and exploring the predictability limits of ensembles of AI forecasts, such as probabilistically-trained AI weather forecast models, e.g., ref. ^29^. However, at current, these models are not available for the February 2020 case study.

Overall, the results of this research highlight the significant potential and progress of data-driven models in improving the accuracy of predicting extreme events such as storm clustering. These findings are based on three representative case studies; thus, systematic evaluation is required to draw more general conclusions regarding the capability of data-driven models in forecasting extreme weather events, especially in terms of the physical consistency of extreme weather events and the associated impact predictions.

These findings align with existing research identifying systematic biases in data-driven midlatitude storm forecasts. AI models significantly underestimated the peak surface winds during Storm Ciarán in November 2023 ^3^ and exhibit a persistent tendency to underestimate cyclone intensity and associated wind fields across the Northern Hemisphere, even at short lead times of less than 10 days^5^. Consequently, a deeper investigation into the physical consistency of midlatitude storms in AI models is necessary. In particular, it is critical to assess whether AI models maintain fundamental physical constraints, e.g., through geostrophic balance. A breakdown in this balance may lead to a weak correlation between MSLP and wind biases, as is here found for the February 2020 storms.

In summary, combining physics-based and data-driven models within hybrid modeling frameworks offers the potential to improve forecasts of extreme weather and climate impacts, including storm clustering events. Such advancements can support the development of more effective tools for local preparedness and early warning systems, thereby mitigating potentially devastating storm impacts.

## Data Availability

The forecasts for all models are available through the WeatherBench2 platform^39^. The ERA5 reanalysis dataset^28^ is freely available through the Copernicus Climate Change Service^40^, as well as through WeatherBench2.
